# Repeat thyroid FNAC: Inter-observer agreement among high- and low-volume centers in Naples metropolitan area and correlation with the EU-TIRADS

**DOI:** 10.3389/fendo.2022.1001728

**Published:** 2022-09-15

**Authors:** Lorenzo Scappaticcio, Pierpaolo Trimboli, Sergio Iorio, Maria Ida Maiorino, Miriam Longo, Laura Croce, Marcello Filograna Pignatelli, Sonia Ferrandes, Immacolata Cozzolino, Marco Montella, Andrea Ronchi, Renato Franco, Mario Rotondi, Giovanni Docimo, Katherine Esposito, Giuseppe Bellastella

**Affiliations:** ^1^ Division of Endocrinology and Metabolic Diseases, University of Campania “L. Vanvitelli”, Naples, Italy; ^2^ Clinic for Endocrinology and Diabetology, Lugano Regional Hospital, Ente Ospedaliero Cantonale, Lugano, Switzerland; ^3^ Istituti Clinici Scientifici Maugeri IRCCS, Unit of Internal Medicine and Endocrinology, Laboratory for Endocrine Disruptors, Pavia, Italy; ^4^ Division of Thyroid Surgery, University of Campania “L. Vanvitelli”, Naples, Italy; ^5^ Pathology Unit, Department of Mental and Physical Health and Preventive Medicine, University of Campania “L.Vanvitelli”, Naples, Italy

**Keywords:** thyroid FNAC, neck ultrasound, ICCRTC, EU-TIRADS, thyroid nodule

## Abstract

Our institution (University Hospital “L. Vanvitelli” - Naples, Italy) is a high-volume (HV) center in Naples metropolitan area and many patients are referred there to repeat thyroid fine-needle aspiration cytology (FNAC) after initial FNAC performed in low-volume institutions (LV). The aims of the study were to 1) examine the inter-observer agreement between HV and LV institutions according to the Italian thyroid cytology system, and 2) explore how the discordant FNAC reports were distributed in the European Thyroid Imaging and Reporting Data System (EU-TIRADS) categories. All consecutive cases of repeat FNAC performed at University Hospital “L. Vanvitelli” from January 2016 to December 2021 were retrospectively reviewed. Fleiss’ kappa (κ) was used to assess the inter-observer agreement, and categorical variables were compared by chi-square testing. P < 0.05 was considered statistically significant. A total of 124 nodules from 124 adults (mean age 49 years; mean maximum diameter 19 mm) were evaluated. Initial FNAC reports at LV were: 4 (3.2%) TIR1c, 64 (51.6%) TIR2, 48 (38.7%) TIR3A, 8 (6.5%) TIR3B, 0 TIR4, 0 TIR5. The overall FNAC reports were significantly different between the LV and HV institutions. At repeated FNAC, cytological diagnosis was unchanged in 64 (51.6%) cases including TIR2 and TIR3A results. A downgraded FNAC diagnosis (i.e., TIR2 vs TIR3A, TIR2 vs TIR3B) was observed in 36 (29%) nodules. An upgraded FNAC diagnosis (i.e., TIR3B vs TIR2, TIR3B vs TIR3A, TIR4 vs TIR3A, TIR5 vs TIR2, TIR5 vs TIR3B) was recorded in 24 (19.4%) nodules. The weighted inter-observer agreement between LV and HV institutions was poor (κ=0.133). Changed FNAC results were significantly (p=0.0023) more frequent in nodules at intermediate/high-risk (i.e., EU-TIRADS 4/5) than in those at no/low risk (EU-TIRADS 2/3) [i.e., 32/48 (66.7%) and 28/76 (36.8%), respectively]. Downgraded FNAC results were significantly more frequent in EU-TIRADS 2/3 (p=0.001) while upgraded FNAC were present only in EU-TIRADS 4/5 (24/24, 100.0%). The inter-observer agreement among LV and HV thyroid services was poor. The EU-TIRADS 4 and 5 categories included all the malignant nodules with FNAC results reclassified as higher risk (i.e., TIR3B-TIR4-TIR5) by the high-volume cytology service.

## Introduction

Neck ultrasonography (nUS) and fine-needle aspiration cytology (FNAC) are the gold standard for diagnosis of thyroid nodules (1).

nUS is the reference exam for an accurate initial management of thyroid lesions, aiming at discriminating the nodules which are worthy of FNAC from those for which FNAC is not needed (1, 2). The Thyroid Imaging Reporting and Data System (TIRADS) by the European Thyroid Association (i.e., EU-TIRADS) is widely used in light of its high performance in stratifying the risk of thyroid nodules (3–6).

Compared to nUS, FNAC remains the gold standard to define the nature of thyroid nodules (1). Similarly to the Bethesda System for Reporting Thyroid Cytopathology (TBSRTC) and UK Royal College of Pathologists (RCPath) system, the Italian Consensus for the Classification and Reporting of Thyroid Cytology (ICCRTC) stratifies the malignancy risk based on FNAC result which dictates the initial treatment of thyroid nodules (i.e., surgery vs conservative approach) (7, 8). These are important decisions which can incur costs and risks (7). Indeed, thyroid surgery is associated to a 1% to 10% risk for long-term sequelae (i.e. hypoparathyroidism and recurrent laryngeal nerve palsy); in addition, with regard to histologic diagnosis, radioactive iodine ablation may increase the risk for secondary malignancies (9–13). Moreover, thyroid cytopathology is deemed to be one of the most subjective domain of diagnosis in pathology practice (14–17), and second opinions are very valuable (8, 18). Therefore, there is usually some extent of uncertainty for the clinical management of thyroid nodules if this is based on the cytologic result alone (1, 8).

At each step of the management of nodules, combining TIRADS evaluation with FNAC result may greatly favour the right therapeutic choices and diminish unnecessary thyroid procedures (2, 19).

However, to our knowledge, limited literature exists on the evaluation of the inter-observer variability of the cytological diagnosis of thyroid nodules according to the three main classification systems (i.e., Bethesda, RCPath, ICCRTC) (20–29). Moreover, few studies were based on raters from institutions with different expertise and case load (21, 29).

In addition, studies that explored the usefulness of the integrated management in the evaluation of thyroid nodules, encompassing the main US-based risk stratification systems and cytology results (30–34), are emerging.

The present study analyses the discordant cytological diagnoses of thyroid nodules from two centers [i.e., a high-volume (HV) and low-volume (LV) institutions, respectively] and the value of a second-opinion diagnosis (SOD) on the cytological material. Specifically, our objectives were: 1) to examine the inter-observer agreement between the HV and LV centers according to the Italian thyroid cytology system, and 2) to explore how the discordant FNAC reports were distributed in the EU-TIRADS categories.

## Methods

### Study design and patients

Our institution (University Hospital “L. Vanvitelli” - Naples, Italy) is a HV center in Naples metropolitan area, and many patients are referred here to repeat FNAC after a first FNAC performed in a LV institution. Here, the request to repeat thyroid FNAC to have a SOD is usually made by a physician outside our institution or by one of our multidisciplinary thyroid team due to concerns about anamnestic, laboratory, nUS and cytological data during nodule follow-up or, less often, prior to a thermal ablation procedure for benign thyroid nodules. In the case of a discordant FNAC result, we recommended thyroid surgery or US follow-up on the basis of the second FNAC cytopathology obtained in our HV center and other clinical factors including EU-TIRADS category, age, comorbidities, patients’ preference.

Adult patients who consecutively referred to our multidisciplinary team to repeat thyroid FNAC from January 2016 to December 2021 were retrospectively identified from those included in the database of the Division of Endocrinology and Metabolic Diseases. This was a multisite analysis since two centers (HV vs LV) examined cytology of the same thyroid nodule. Patients were recruited from Naples metropolitan areag comprising 10 community-based practices and 10 cytologists across 26 smaller towns (i.e., LV center with a smaller case load, less complex cases and without a multidisciplinary team).

Cases were included whether: a) the second FNAC diagnosis was achieved by HV cytologists blind of the previous LV report and 6 to 18 months later from the first FNAC; b) HV FNAC samples were independently evaluated by two observers with full concordance for the final result; c) nodules could be classified according to the EU-TIRADS by two endocrinologists reviewing at least four clear B-Mode US images blind of FNAC reports.

Patients were excluded if they had: a) cytologically indeterminate nodules without histology; b) nodules showing non-diagnostic (i.e., TIR1) cytology; c) cytologically benign results in our center with less than 3 years of US stability; d) nodules with suspicious of malignancy or malignant cytology without final diagnoses determined by surgery; e) incomplete data (i.e., hormonal and antibodies profile) and positive serum calcitonin.

### Thyroid ultrasonography

At our center nUS images were obtained by the same experienced operator (S.I., with 35 years of clinical experience in performing nUS) for the evaluation of thyroid nodules before the US-guided FNAC procedure by an ultrasound device (MyLab^™^Six, Esaote) with a 7-14 MHz wide band linear transducer. The color gain was adjusted so that artifacts were prevented. The examination of ultrasonographic features of thyroid nodules, along with thyroid vascularity and volume, were systematically conducted for patients presenting for thyroid assessment to our Division.

When reviewing the US images on digital format, two endocrinologists (G.B. and L.S. with 20 and 8 years of clinical experience, respectively, in performing and evaluating thyroid US) assessed the thyroid nodules by using the criteria of EU-TIRADS, being unaware of nodule’s cytopathology and histopathology, of laboratory and imaging results. In case of disagreeing US categorization, a consensus with the help of a third senior reviewer (P.T.) (also unaware of pathology or any other patient data) was reached.

### Thyroid nodule pathology

At our center US-guided FNAC was routinely performed by using a 23- gauge needle using a conventional method, and at least two needle passes were performed for each nodule. In all cases, direct air-dried smears were made after the FNAC procedure, then stained by using the May-Gruenwald-Giemsa (MGG) method. All the available slides from each case were reviewed. All US-guided procedures were performed by two faculty operators (I.C. and S.I., with 20 and 35 years of clinical experience, respectively, in performing thyroid FNAC).

All cytology specimens were reviewed by two thyroid cytopathologists (I.C. and M.M., with 20 and 10 years of clinical experience in thyroid cytopathology). In case of disagreeing FNAC subcategorization, a consensus with the help of a third senior reviewer (R.F.) (also unaware of pathology or any other patient data) was reached. At all times, the two pathologists (I.C., M.M.) were unaware of both the previous cytopathologic diagnoses made by LV pathologists and histopathologic diagnoses made by other pathologists of our Institution. They also were unaware of demographics and clinical data, including US features of thyroid nodules.

At the two centers the cytologic diagnoses were reported according to the five subcategories of the revised ICCRTC (7). At our center all cytopathology reports were collected, blinded, assigned a new study identification number to allow comparison with LV diagnoses. The final pathology (i.e., histology of the thyroid nodule after surgery) was made according to the World Health Organization (WHO) book on endocrine tumors classification (35).

Unchanged FNAC reports corresponded to the same diagnoses between LV and HV centers, and these also encompassed the scenario TIR1c in the LV center vs TIR2 in the HV center. Downgraded FNAC diagnoses corresponded to “better” results obtained in the HV vs the LV center (i.e., TIR2 vs TIR3A, TIR2 vs TIR3B). Conversely, upgraded FNAC diagnoses corresponded to “worst” results obtained in the HV vs the LV center (i.e., TIR3B vs TIR2, TIR3B vs TIR3A, TIR4 vs TIR3A, TIR5 vs TIR2).

### Statistical analysis

Continuous variables were described as median and interquartile range (IQR). Categorical variables were presented as number (percentage). For both cytological and US analyses the interobserver agreement was evaluated by Fleiss’ kappa (κ), where the κ value means the strength of agreement and is interpreted as follows: 0–0.2, poor; 0.2–0.4, fair; 0.4–0.6, moderate; 0.6–0.8, good; 0.8–1.0 very good. Categorical variables were compared by Chi-square testing, so that the distribution of FNAC results at our center was compared to data from LV center. Each of the EU-TIRADS categories was analyzed to determine its association with a benign or malignant diagnosis. Statistical significance was defined as a *p* value < 0.05. Statistical analysis was performed by MedCalc software version 9 (Mariakerke).

## Results

**Figure 1:**
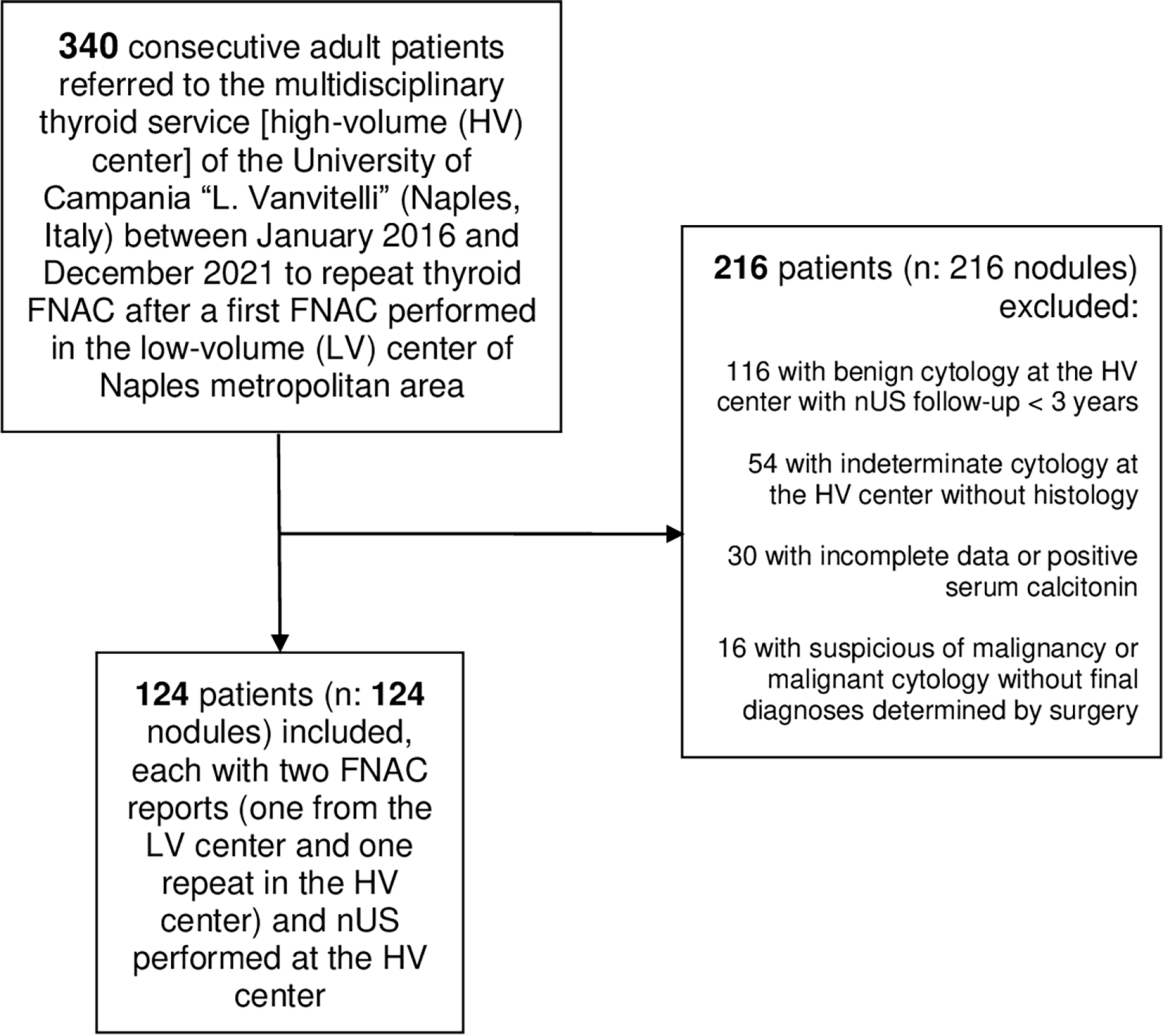
Flowchart of patients’ selection nUS, neck ultrasound; FNAC, fine needle aspiration cytology.

Three hundred and forty consecutive patients were referred to our HV center to repeat thyroid FNAC after a first FNAC performed in the LV center of Naples metropolitan area. After applying our exclusion criteria, we finally included in the study 124 nodules from 124 adults (**Figure 1**). One hundred sixteen patients were female (93.5%), and eight patients were male (6.5%). Median age was 49 years (43-55 years). The median nodule’ s maximal dimension was 19 mm (13-23mm). Of the 124 thyroid nodules, 100 (80.6%) were benign [of which 40 (40.0%) underwent surgery] and 24 (19.4%) were malignant. All cancers were papillary carcinoma [16 (66.7%) of 24 conventional variants, of which four with 20% of tall cells, and eight (33.3%) follicular-variant]. Median maximal dimension of malignant thyroid nodules was 14.5 (11–16). Benign nodules were distributed in the following categories of EU-TIRADS: 24 (24%) in 2; 52 (52%) in 3; 20 (20%) in 4; 4 (4%) in 5. Twelve malignant nodules fell into EU-TIRADS 4 and the remaining twelve into EU-TIRADS 5. The characteristics of our patients are shown in **Table 1**.

**Table 1:** Main characteristics of participants in the study (n: 124).

Characteristics	
Age at diagnosis, years (IQR)	49.0 (43-55)
Females/Males (n)	116/8
Nodules	
• maximal dimension, mm (IQR)	19 (13-23)
Benign nodules/Malignant Nodules, n (%)	100/24 (80.6/19.4)
• benign with surgery	40/100 (40.0)
Malignant nodules	
• maximal dimension, mm (IQR)	14.5 (11-16)
• cPTC, n (%)	16* (66.7)
• fvPTC, n (%)	8 (33.3)
EU-TIRADS categories: benign nodules, n (%)	
• 2	24 (24)
• 3	52 (52)
• 4	20 (20)
• 5	4 (4)
EU-TIRADS categories: malignant nodules, n (%)	
• 2	0
• 3	0
• 4	12 (50)
• 5	12 (50)

IQR, interquartile range; nUS, neck ultrasound; mm, millimeter; cPTC, classic papillary thyroid cancer; fvPTC, follicular-variant papillary thyroid cancer; EU-TIRADS, European Thyroid Imaging and Reporting Data System.

*four cases had 20% of tall cells.

As shown in **Table 2**, initial FNAC reports at the LV center were: 4 (3.2%) TIR1c, 64 (51.6%) TIR2, 48 (38.7%) TIR3A, 8 (6.5%) TIR3B, 0 TIR4, 0 TIR5. Repeat FNAC reports at the HV center were: 0 TIR1c, 92 (74.2%) TIR2, 8 (6.5%) TIR3A, 8 (6.5%) TIR3B, 4 (3.2%) TIR4, 12 (9.7%) TIR5. The overall FNAC reports were significantly different between the LV and HV institutions.

**Table 2:** Distribution of thyroid FNAC results of the 124 included nodules as per diagnostic subcategory in the two centers (i.e. LV center vs HV center).

ICCRTC subcategories	LV (first FNAC)	HV (repeat FNAC)	p value
• TIR1c	4	0	
• TIR2	64	92	
• TIR3A	48	8	
• TIR3B	8	8	
• TIR4	0	4	
• TIR5	0	12	
*total*	124	124	0.0001

FNAC, fine-needle aspiration cytology; ICCRTC, Italian Consensus for the Classification and Reporting of Thyroid Cytology; LV, low-volume; HV, high-volume.

Statistical significance as a p value < 0.05.

**Figure 2:**
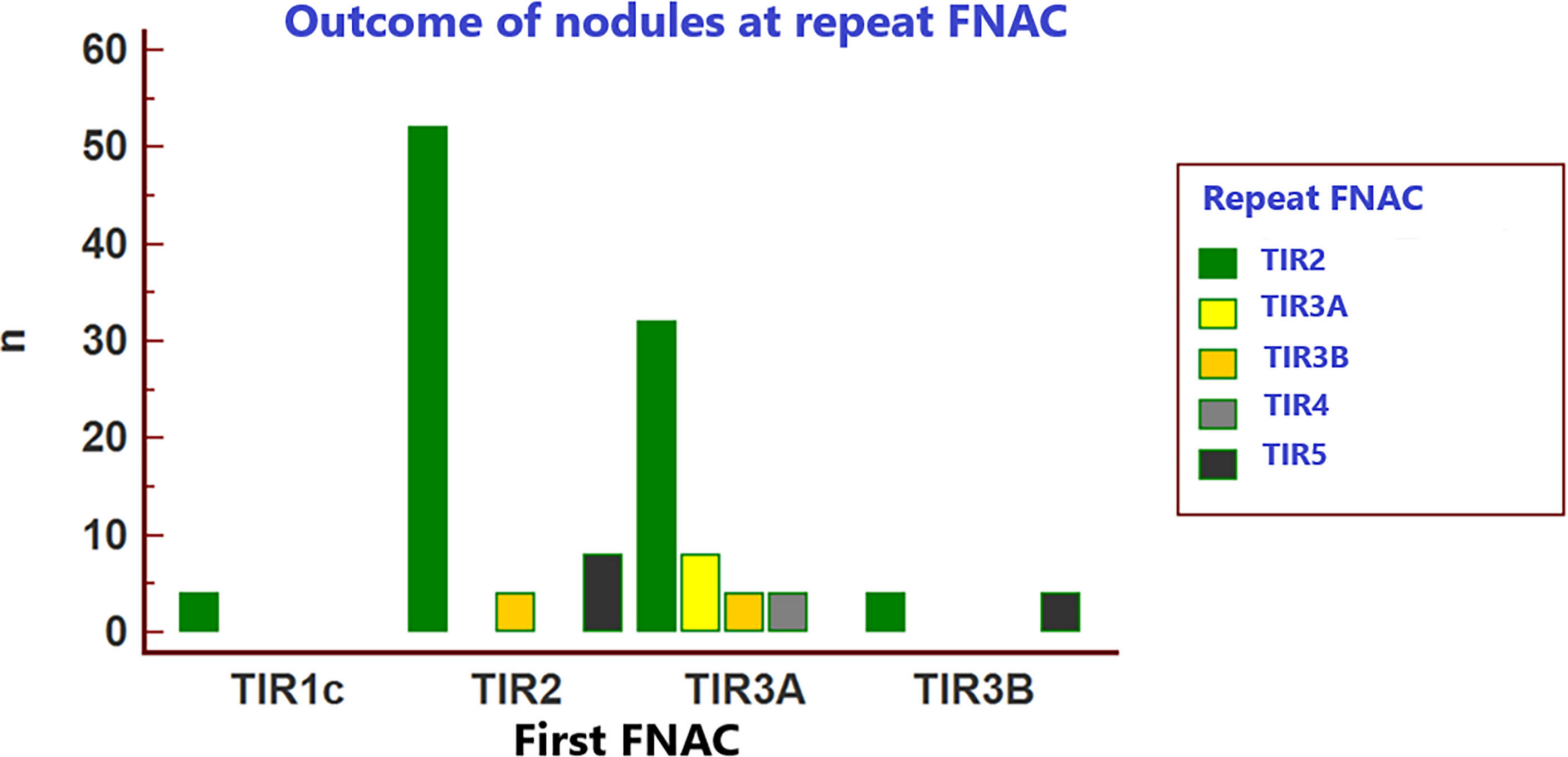
Outcome of the 124 included nodules at repeat FNAC.

**Table 3** and **Figure 2** resume the outcome of the initial FNAC reports when in the HV center cytology was repeated on each nodule. At repeated FNAC, cytological diagnosis was unchanged in 64 (51.6%) cases, including 56 TIR2 and 8 TIR3A results. A downgraded FNAC diagnosis (i.e., TIR2 vs TIR3A, TIR2 vs TIR3B) was observed in 36 (29%) nodules, including: 32 cases TIR2 vs TIR3A and four TIR2 vs TIR3B. An upgraded FNAC diagnosis (i.e., TIR3B vs TIR2, TIR3B vs TIR3A, TIR4 vs TIR3A, TIR5 vs TIR2, TIR5 vs TIR3B) was recorded in 24 (19.4%) nodules, including: four cases TIR3B vs TIR2, four TIR3B vs TIR3A, four TIR4 vs TIR3A, eight TIR5 vs TIR2, four TIR5 vs TIR3B. The weighted inter-observer agreement between LV and HV institutions was poor (κ=0.133).

**Table 3:** Inter-observer agreement of thyroid FNAC results of the 124 included nodules between the two centers (i.e. LV center vs HV center) according to the ICCRTC.

LV (first FNAC)							
HV (repeat FNAC)	TIR1c	TIR2	TIR3A	TIR3B	TIR4	TIR5	total, n (%)
• TIR1c	0	0	0	0	0	0	0
• TIR2	4	52	32	4	0	0	92 (74.2)
• TIR3A	0	0	8	0	0	0	8 (6.5)
• TIR3B	0	4	4	0	0	0	8 (6.5)
• TIR4	0	0	4	0	0	0	4 (3.2)
• TIR5	0	8	0	4	0	0	12 (9.7)
*total, n (%)*	4 (3.2)	64 (51.6)	48 (38.7)	8 (6.5)	0	0	124
*weighted κ*							0.133

FNAC, fine-needle aspiration cytology; LV, low-volume; HV, high-volume; ICCRTC, Italian Consensus for the Classification and Reporting of Thyroid Cytology.

κ value of 0–0.2 indicates a poor inter-observer agreement.

**Table 4** analyzes the discordant FNAC results between the two centers according to the EU-TIRADS categories and final histology. Changed FNAC results were significantly more frequent in nodules at intermediate/high-risk (i.e., EU-TIRADS 4/5) than in those at no/low risk (EU-TIRADS 2/3) [i.e., 32/48 (66.7%) and 28/76 (36.8%), respectively, p=0.0023]. Downgraded FNAC results were significantly more frequent in EU-TIRADS 2/3 [i.e., 28/36 (77.7%) versus 8/36 (22.3%) in EU-TIRADS 4, p=0.001] while upgraded FNAC results were present only in EU-TIRADS 4/5 (12/24, 50.0% in EU-TIRADS 4 and 12/24, 50% in EU-TIRADS 5). A 100% cancer prevalence (24/24) was observed in the upgraded FNAC cases. The inter-observer agreement in classifying nodules according to the EU-TIRADS was good (k-value of 0.7, p < 0.002).

**Table 4:** Analysis of discordant FNAC results between the two centers according to the EU-TIRADS categories and final histology.

	*total*	*EU-TIRADS categories*				*Histology*		
		*2*	*3*	*4*	*5*	Benign*	*Malignant*
***Unchanged FNAC results, n (%)***	64 (51.6)	20	28	12	4	64 (51.6)	0
***Discordant FNAC results, n (%)***	60 (48.4)						
• downgraded	36 (29)	4	24	8	0	36 (29)	0
• upgraded	24 (19.4)	0	0	12	12	0	24
*total*	124	24	52	32	16	100	24

FNAC, fine-needle aspiration cytology; EU-TIRADS, European Thyroid Imaging and Reporting Data System.

Unchanged FNAC result: same diagnoses between low-volume (LV) and high-volume (HV) centers.

Downgraded FNAC results: “better” results obtained in the HV vs the LV center (i.e., TIR2 vs TIR3A, TIR2 vs TIR3B). Upgraded FNAC results: “worst” results obtained in the HV vs the LV center (i.e., TIR3B vs TIR2, TIR3B vs TIR3A, TIR4 vs TIR3A, TIR5 vs TIR2).

*Benignity was determined in 40 nodules at histology, while in 60 nodules at cytology plus US stability at the follow-up of not less than three years.

Malignancy included 16 cases of classic papillary thyroid cancer (PTC) (four cases with a 20% of tall cells) and 8 follicular-variant PTCs.

## Discussion

In this real-practice and observer-blinded study we finally included 124 nodules, of which each was first cytologically analyzed in the LV center, then a second FNAC was performed in our HV thyroid service. In our center EU-TIRADS was adopted to stratify the risk of malignancy of each nodule based on US features (3, 4). We found a PTC prevalence of about 20%, that was greater than that usually reported as consequence of the high selection of patients in our tertiary care service (1).

Firstly, we demonstrated that the overall FNAC results according to the Italian thyroid cytology system were significantly different between the two centers: indeed, as already noted in some studies according to other internationally recognized systems for reporting thyroid cytology (20, 21), we observed that the cytopathologists of the HV center tended to score lower the benign nodules and higher those with final diagnosis of malignancy. Perhaps, this was due to the extensive expertise of our thyroid cytopathologists to more easily detect both the benign and malignant cases, compared with operators that work in LV services where thyroid FNAC occupies only a small place in the daily routine. One other possible explanation of this result consists in the repetition of FNAC (and not the revision of the slides), whereby aspirators could have produced material from different points of the nodule or simply more material.

Specifically, we found a poor inter-rater reproducibility of thyroid FNAC results between the two centers when using the Italian thyroid cytology system. In fact, almost half of the patients were found to have a discordant FNAC result when the nodule was analysed at the HV center and the unchanged FNAC reports mostly included benign cytology diagnoses (i.e., ~ 90% were TIR2).

However, it is noteworthy that about one out of five benign cytology diagnoses by the LV center were upgraded by the HV center: indeed, four cases became high-risk indeterminate (i.e., TIR3B) lesions and eight cases were reported as malignant (i.e., TIR5) lesions by the HV center, leading to the adoption of surgery for these cases as suggested by the ICCRTC (6) and showing a PTC diagnosis in all cases.

Regarding the low-risk indeterminate subcategory of the ICCRTC (i.e., TIR3A) only about one out of five cases were reported in the same subcategory by the HV center, while the remaining three fifths and one fifth were respectively reclassified as benign (i.e., TIR2) and with higher risk (i.e., TIR3B or TIR4) by the HV center. In this respect, the eight cases that upgraded from TIR3A of the LV center to subcategories requiring surgery (i.e., TIR3B or TIR4) at the HV center were submitted to thyroidectomy due to recommended actions (6) and in all cases a diagnosis of PTC was made. Conversely, at the HV center a conservative approach was tendentially adopted for the TIR3A cases that downgraded to TIR2, and when surgery was chosen this approach was not driven by the FNAC result.

With regard to the TIR3B results by the LV center, half was reclassified as TIR2 and half as TIR5 by the HV center. In the four TIR3B cases upgraded to TIR5 subcategory in the HV center the surgical management decision did not change in compliance with the recommended actions of the ICCRTC (7) and four corresponding malignancies were detected. By contrast, the four TIR3B cases downgraded to TIR2 subcategory in the HV center were conservatively managed: revision by our cytopathologists of the slides by the LV center confirmed a benign cytology diagnosis in all cases and a US stability of these four nodules has been demonstrated to date.

The above findings are in line with the study by Cibas et al. (21), which according to TBSRTC, indicated that, compared with a community center that have lower volumes and sub-specialty, in academic centers the cytopathologists with experience in thyroid cytopathology are more likely to make a definitive interpretation (i.e., more benign and malignant diagnoses) and fewer indeterminate diagnoses. Moreover, according to the little data available, despite the vast majority of the studies have been done in academic or tertiary care centers, it has been reported that inter-observer agreement is relatively poor in indeterminate diagnoses while it is moderate or good among raters with thyroid cytopathology expertise in benign and malignant cytology diagnoses (21, 28). We can speculate that the poor inter-observer reproducibility of indeterminate FNAC results may be also due to the not very clear category criteria or the pathologist’s training (8, 16, 25). In addition to the study by Cibas et al. (21), the study by Le et al. (29) explored the distribution of thyroid FNAC results in a community center. However, inter-rater agreement was not calculable since the included nodules were not also examined by an academic or tertiary center (29).

In our cohort, compared with nodules with unchanged FNAC results, nodules with discordant FNAC results more frequently fell into the intermediate and high-risk (i.e., EU-TIRADS 4 and 5) US categories than into no and low risk (EU-TIRADS 2 and 3) categories. Specifically, downgraded FNAC results were significantly more frequent in EU-TIRADS 2/3; on the other hand, nodules with upgraded FNAC results, which had a 100% cancer prevalence, only fell into EU-TIRADS 4 and 5 (i.e., half in EU-TIRADS 4 and half in EU-TIRADS 5). These findings are in line with the emerging literature supporting a key role of the TIRADS in the management of thyroid nodules also after FNAC: namely that suspicious sonographic features are optimal predictors of malignancy in thyroid nodules with previous benign or indeterminate cytology (1, 30–34).

In our study almost half of patients resulted in changes in their management decisions: about 30.0% of discordant cases were not oriented to surgery because of the downgraded FNAC result at the HV center; while about 20.0% of patients had appropriate oncologic thyroid resection as a result of the repeat FNAC. Not repeating FNAC in the HV center would have potentially missed or delayed diagnosis of PTC in about 20.0% of cases. Therefore, in accordance with the study by Gerhard et al. (18), our study demonstrates that a SOD on pathological material by a HV center improves the clinical management of thyroid nodules when the first evaluation has been made in a LV center. In line with other studies, this is a valuable strategy especially for US–pathology mismatched nodules (31, 36–38). The strengths of our study are the following: 1) to our knowledge, this is the first study regarding the evaluation of the inter-observer agreement in the FNAC subcategories of the Italian thyroid cytology system between two centers with different expertise and case load. The other two studies using the ICCRTC reported in the literature explored the inter-oberver agreement among highly experienced cytologists (27) and in the indeterminate categories among raters with various degree of training in thyroid cytopathology (28), respectively; 2) in addition to the current literature we also evaluated how the discordant FNAC reports were distributed in the EU-TIRADS categories and the value of the SOD on pathological material of repeat FNAC when the first FNAC was performed in a LV center.

The limitations of our study also should be discussed. First, this study is limited mainly by the retrospective design. There may be a selection bias in the patients that had repeat FNAC, influenced by the fact that the decision to perform a second FNAC in our HV center was on nodules with initial benign or indeterminate (mostly low-risk indeterminate) cytology and also made by an external physician. Indeed, a second FNAC was not performed in nodules with previous suspicious of malignancy (i.e., TIR4) and malignant (i.e., TIR5) reports. Second, not all the cytologically benign results of the HV center were confirmed after surgery. However, less than 50% of benign FNAC at the HV center had only US stability at the long-term follow-up as the best mark of benignity and our aims were not to derive conclusions on FNAC accuracy in predicting malignancy comparing HV versus LV centers. Still, a 3 year follow-up is a good time frame to detect US-features variations (31). Third, discordant FNAC results were not evaluated according the thyroid nodule sizes. However, for upgraded FNAC diagnoses mean nodule size was small and maximal dimension was not more than 27 mm (i.e., in the HV center only 4/60 nodules with changed FNA report was more than 20 mm). Fourth, no data regarding the inter-observer agreement in the non-diagnostic (i.e., TIR1) category was possible, since we decided to include only conclusive cytological diagnoses, and non-diagnostic-cystic (i.e., TIR1C) category was not obtainable in our center as TIR1C diagnosis requires that the cytopathologist is aware of the dominant cystic composition of the nodule. Fifth, outside of Italy pathologists rarely use the ICCRTC System, so that our report does not allow to make comparison with other systems for reporting thyroid cytology. Sixth, our results reflect the scenario of high- and low-volume thyroid services in Naples metropolitan area, which can be different from that of other Italian metropolitan areas and worldwide.

## Conclusion

Our findings suggest that FNAC result varies among institutions with different expertise and case load, and this may affect its utility in the diagnostic workup of thyroid nodules. Specifically, one FNAC could not be sufficient for benign/malignant thyroid nodule diagnosis when the first FNAC is pursued in a LV center. A comprehensive analysis of initially FNAC-proven benign/indeterminate thyroid nodules by EU-TIRADS guidelines is useful for the interpretation of cytology results. It is important that cytopathologists and clinicians are aware of the poor interobserver reproducibility of the various cytologic subcategories among HV and LV centers and that they use this information in their clinical decision making. If further validated, such findings could call into question the utility of FNAC outside of tertiary centers and have profound downstream consequences in the management of thyroid nodules.

## Data availability statement

The original contributions presented in the study are included in the article/supplementary material. Further inquiries can be directed to the corresponding author.

## Ethics statement

The studies involving human participants were reviewed and approved by The ethics committee of the University Hospital “L. Vanvitelli” (Naples, Italy). The patients/participants provided their written informed consent to participate in this study.

## Author contributions

LS, PT, and GB designed and conceptualized the study, analyzed the data and drafted the manuscript for intellectual content. All the co-authors interpreted the data and revised the manuscript for intellectual content. All authors contributed to the article and approved the submitted version.

## Conflict of interest

The authors declare that the research was conducted in the absence of any commercial or financial relationships that could be construed as a potential conflict of interest.

## Publisher’s note

All claims expressed in this article are solely those of the authors and do not necessarily represent those of their affiliated organizations, or those of the publisher, the editors and the reviewers. Any product that may be evaluated in this article, or claim that may be made by its manufacturer, is not guaranteed or endorsed by the publisher.
